# A review of literature and meta-analysis of one-puncture success rate in radiofrequency thermocoagulation with different guidance techniques for trigeminal neuralgia

**DOI:** 10.1186/s40001-022-00758-0

**Published:** 2022-08-06

**Authors:** Zhengming Wang, Xu Su, Yin Yu, Zhijun Wang, Kai Li, Yufei Gao, Yu Tian, Chao Du

**Affiliations:** 1 Department of Neurosurgery, The Third Hospital of Jilin University & China-Japan Union Hospital, No. 126, Xiantai Street, Changchun, 130033 China; 2 Department of Pediatric Surgery, The First Hospital of Jilin University, Changchun, China; 3 Department of Anesthesia, The Third Hospital of Jilin University & China-Japan Union Hospital, Changchun, China

**Keywords:** Trigeminal neuralgia, Puncture accuracy, One-puncture success rate, Radiofrequency thermocoagulation, Gasserian Ganglion, Ablative interventions

## Abstract

**Objectives:**

Radiofrequency thermocoagulation (RFT) is a type of Gasserian ganglion-level ablative intervention that is used for the treatment of trigeminal neuralgia. Guidance technologies are used to assist in the cannulation of the foramen ovale (FO) or foramen rotundum (FR) target. We conducted a systematic review to assess the value of different guidance technologies for RFT.

**Methods:**

We searched PubMed, Embase, the Cochrane database, Web of Science, and PROSPERO for studies published from January 2005 until December 2020. Randomized or nonrandomized comparative studies and nonrandomized studies without internal controls were included. The Cochrane Risk of Bias Tool and the nonrandomized studies of interventions-I tool were used to assess individual study characteristics and overall quality.

**Results:**

Our query identified 765 publications, and we were able to analyze 11 studies on patients suffering from trigeminal neuralgia. Only one study involved randomized controlled trials, whereas the others featured nonrandomized designs, predominantly before-and-after comparisons. Most of them were observational studies. A total of 222 participants were included, with a median number (range) of 20 (3–53) participants. The objective response rate (ORR) of the one-puncture success rate of RFT using puncture guidance for trigeminal neuralgia was 92% [95% CI (0.79–1), *P* < 0.001]. Statistically significant differences were observed in the cannulation and operation times between the guided and manual puncture groups (*P* < 0.001).

**Conclusions:**

RFT with puncture guidance technology has an absolute advantage in puncturing the foramen ovale or foramen rotundum.

## Introduction

Trigeminal neuralgia (TN) is a one of the most painful conditions afflicting humans [1]. Percutaneous procedures, including radiofrequency thermocoagulation (RFT) [2], percutaneous balloon compression (PBC) [3], and glycerol rhizolysis (GR) [4], should be the preferred choice for TN with no vascular contact [5]. RFT is a type of Gasserian ganglion-level ablative intervention [6] that was first developed by Réthi in 1913 [7]. RFT applies radiofrequency heat lesions to block the trigeminal ganglion through puncture of the foramen ovale or the foramen rotundum.

In RFT, the target neuron in the ganglion is located via sensory stimulation to avoid an inadvertent neurolytic block of the unaffected branches [8]. However, manual puncture procedures always involve multiple punctures and adjustments of stimulation, which increase the suffering of patients and could cause serious complications [9–12]. Accurate cannulation of the FO target even to the Gasserian ganglion (GG) target is an important part of determining the success or failure of the procedures. Techniques and applications used in solving this problem include neuronavigation [13], 3D templates [14], and stereotactic guidance [15, 16]. Neuronavigation is a technology which provides visual puncture guidance during the cannulation. 3D templates technology provides a cannulation trajectory through a 3D printed template placing on the patient’s face. Stereotactic provides trajectory through a frame on the head. Surgeons choose different guidance techniques according to their personal preferences. However, there is no article that gives an overall evaluation of these technologies. Here, we review the one-puncture success rate and efficacy to assess the value of different guidance methods for RFT.

## Materials and methods

This systematic review was conducted in accordance with the PRISMA guidelines, the checklist for which was completed [17].

### Protocol and registration

This study was registered in the PROSPERO database (Registration Number: CRD42020201479).

### Search strategy

We searched for English articles in PubMed, Embase, the Cochrane database, Web of Science, and PROSPERO that were published from January 1, 2005, to December 31, 2020. The databases were queried using the following search terms: “trigeminal neuralgia,” “radiofrequency thermocoagulation,” “neuronavigation,” “3D printing,” and “stereotactic.” The search was limited to articles on human studies. We demonstrated the search strategy applied using the PubMed search engine as an example. We also used the reference lists of relevant articles to search for articles.

### Inclusion and exclusion criteria

Studies with TN patients treated by radiofrequency thermocoagulation at the Gasserian ganglion were enrolled in the study. RFT with neuronavigation, 3D template, or stereotactic guidance was directed to the Gasserian ganglion, and data on the puncture success rate of each were collected. Any type of comparator was eligible, including manual puncture, fluoroscopy guidance, or none.

### Selection of studies and data extraction

Randomized trials and nonrandomized studies including those without an internal control group (cohort or case series) were included. No publication date restrictions were imposed. Two authors independently assessed titles and abstracts retrieved via database searches (WZM, WZJ), as well as full texts of potentially relevant studies. Any discrepancies between the authors were resolved by the involvement of a third author (LK). Two authors (WZM, WZJ) independently extracted the following information from each study: name of the first author; year of publication; study design; comparator; inclusion and exclusion criteria; number of participants; follow-up period; guidance method; temperature and time of RFT; complications; and results on puncture accuracy.

### Outcome measures

The primary outcome measure was the one-puncture success rate. Secondary outcomes included puncture time and operation time to describe the puncture accuracy at the puncture location.

### Statistical analysis

Stata 16.0 was used for meta-analysis. The heterogeneity among the results of the included studies was analyzed using the *χ*^2^ test (the test level was *α* = 0.1), and the magnitude of the heterogeneity was quantitatively judged in conjunction with *I*^2^. If no statistical heterogeneity was found between the results of each study, the fixed-effects model was used for the meta-analysis. However, if statistical heterogeneity was observed between the results of each study, the random-effects model was used for meta-analysis. Obvious clinical heterogeneity was evaluated by descriptive analysis. The test level of the meta-analysis was set to *α* = 0.05. Begg’s and Egger’s methods were used to test for publication bias.

### Risk of bias

Two authors with formal training in assessing medical literature according to the principles of evidence-based medicine (WZM and YY) assessed the risk of bias of included randomized controlled trials (RCTs) and non-RCTs using the Cochrane Risk of Bias Tool [18] and the nonrandomized studies of interventions-*I* tool, respectively [19]. Discrepancies were resolved by the third author (LK).

## Results

### Study selection

**Fig. 1:**
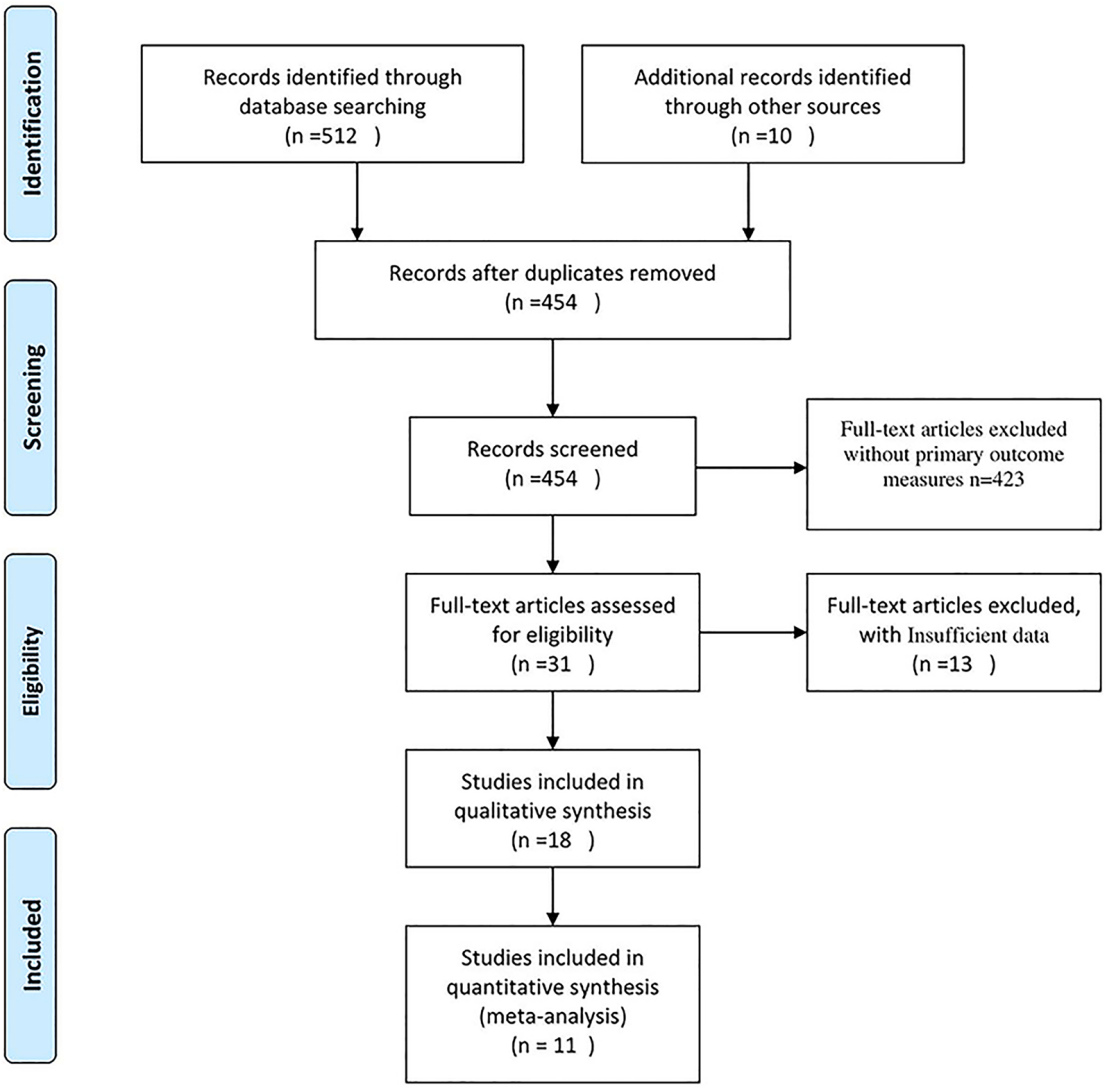
Study flowchart

We retrieved 765 records via an electronic database search and 10 records via searches of reference lists, citations, and other reviews. After deduplication, 545 unique records were screened against the eligibility criteria, of which the full texts of 31 manuscripts were analyzed. Ultimately, 11 manuscripts were analyzed in this systematic review (Fig. 1). The characteristics of the included studies are described in Table 1. The studies involved various designs, including eight retrospective cohort studies, 1 RCT, one case report, and one case series.

**Table 1:** Description of included studies

Author, year	Study design	No. of participants	Guidance method	Temperature and time of RFT	Primary outcome measures	Target point	Refs.
Wang et al., 2019	Retrospective study	17	3D template	60 ℃ for 120 s, 65 ℃ for 120 s, and 70 ℃ for 120 s	One puncture success rate	Foramen rotundum	[21]
		21	Manual puncture	60 ℃ for 120 s, 65 ℃ for 120 s, and 70 ℃ for 120 s	One puncture success rate	Foramen rotundum	
Zhao et al., 2018	Retrospective study	53	3D template	65 ℃, 70 ℃, 75 ℃, 80 ℃ in turn for 90 s	Operation time	Foramen ovale	[30]
		64	Manual puncture	65 ℃, 70 ℃, 75 ℃, 80 ℃ in turn for 90 s	Operation time	Foramen ovale	
Zhang et al., 2018	Retrospective study	32	3D template	65 ℃, 70 ℃, 75 ℃, 80 ℃, 85 ℃ in turn for 90 s	One puncture success rate	Foramen rotundum	[23]
		20	Manual puncture	65 ℃, 70 ℃, 75 ℃, 80 ℃, 85 ℃ in turn for 90 s	One puncture success rate	Foramen rotundum	
Deng et al., 2017	Retrospective study	11	3D template	65 ℃, 70 ℃, 75 ℃, 80 ℃ in turn for 90 s	One puncture success rate	Foramen Ovale	[24]
Lu et al., 2015	Randomized controlled trial	30	3D template	60 ℃, 65 ℃, 70 ℃ in turn for 60 s	One puncture success rate	Foramen ovale	[20]
Guo et al., 2019	Case series	4	3D CT + Frame stereotactic	70 ℃ in the V1 and 75 ℃ in the V2 and V3, the time was not mentioned	One puncture success rate	Foramen ovale	[22]
Mandat et al., 2009	Retrospective study	3	Frameless stereotactic	70 ℃ for 60 s	One puncture success rate	Foramen ovale	[27]
Bale et al., 2006	Retrospective study	15	Frameless stereotactic	N/A	One puncture success rate	Foramen ovale	[28]
Zheng et al., 2018	Retrospective study	44	CT navigation	65 ℃, 70 ℃, 75 ℃, 80 ℃, 85 ℃ in turn for 60–90 s	Operation time	Foramen ovale	[29]
Lin et al., 2011	Retrospective study	42	CT navigation	60–70 ℃ for 60–90 s	One puncture success rate	Foramen ovale	[26]
Chen et al., 2013	Case report	3	Electromagnetic navigation	Temperature ranged from 65 to 80 ℃ for 300 s	One puncture success rate	Foramen ovale	[25]

### Risk of bias and quality of evidence in included studies

**Fig. 2:**
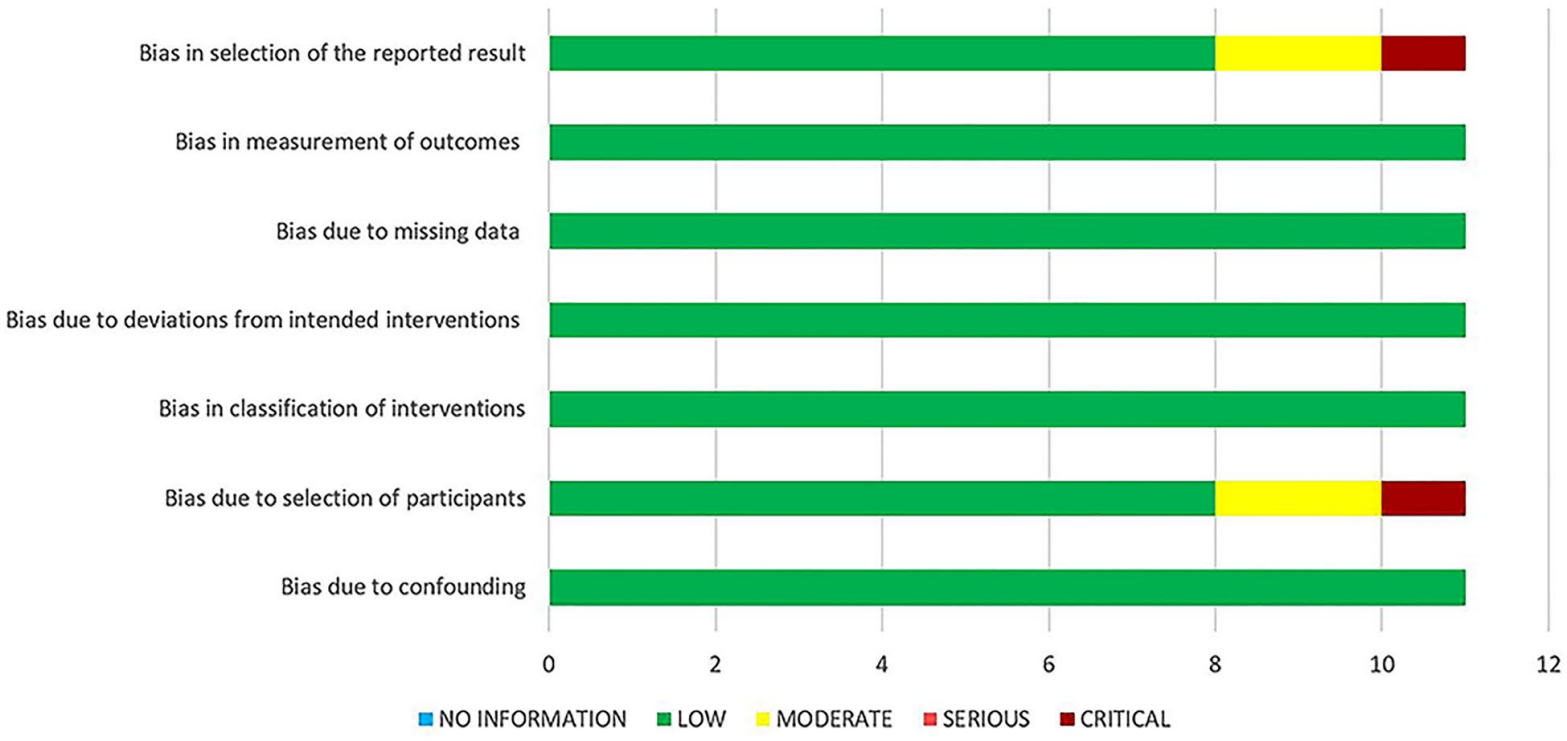
Overall risk of bias summary of nonrandomized studies

Only one RCT was included in the study [20]. The random-sequence generation domain was low for a particular randomization method. The allocation concealment domain had a high risk of bias, because the surgeon knew the patients’ histories, since double-or triple-blinding is difficult to achieve in surgical observation studies. Since we used objective evaluation indicators, we believed that it would not affect the results. We defined a low risk of bias for the domains “blinding of participants and personnel” and “blinding of outcome assessment” in the study. A low risk of bias was defined for the domain “incomplete outcome data.” Since no patients were lost to follow-up, the selective reporting bias was low. We further defined a low risk for other biases. The nonrandomized studies of the interventions-*I* tool were used to assess the risk of bias of the other 10 studies. The results of this assessment are shown in Fig. 2.

### Meta-analysis outcomes

**Fig. 3:**
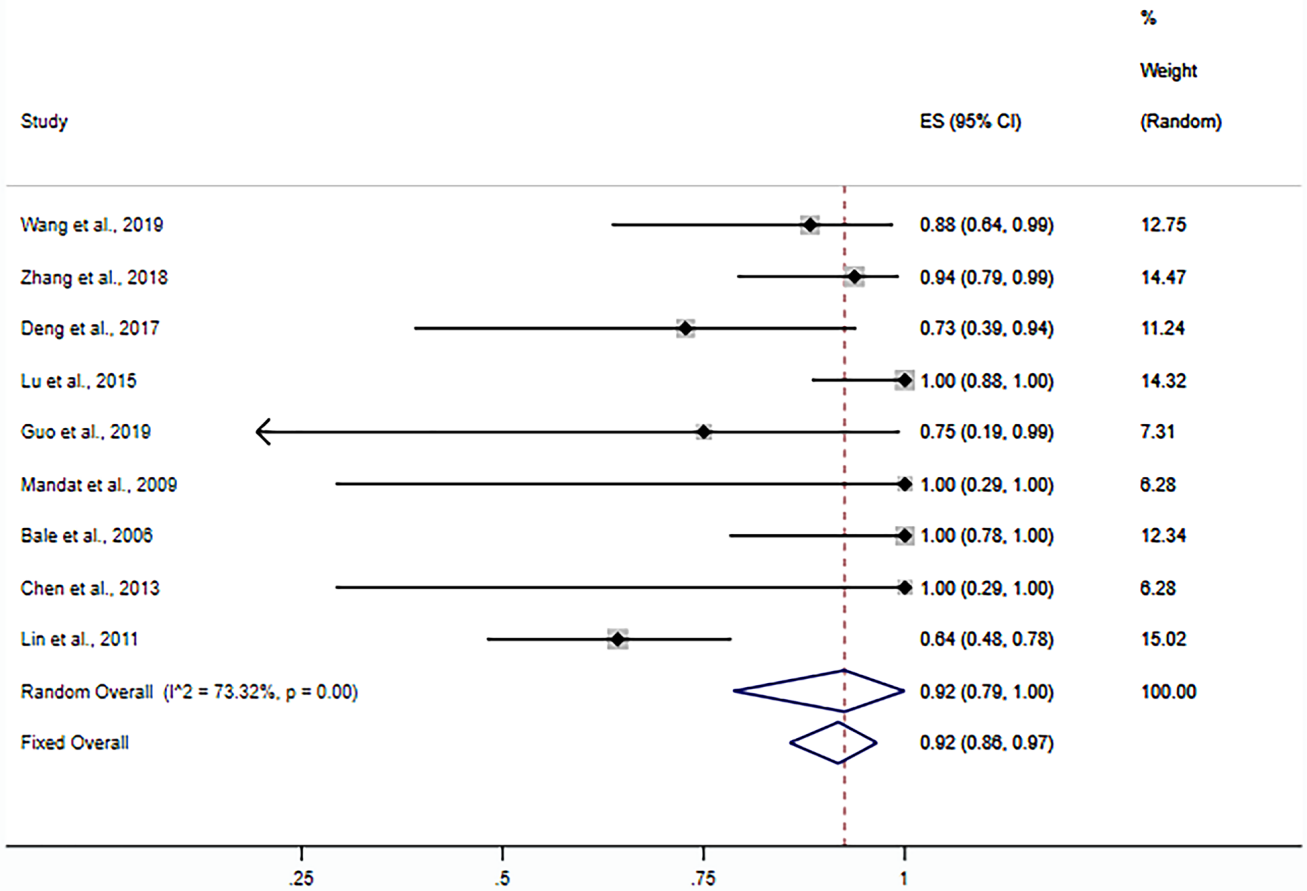
Forest plot of the ORR of the one-puncture success rate using puncture guidance in radiofrequency thermocoagulation for TN

**Fig. 4:**
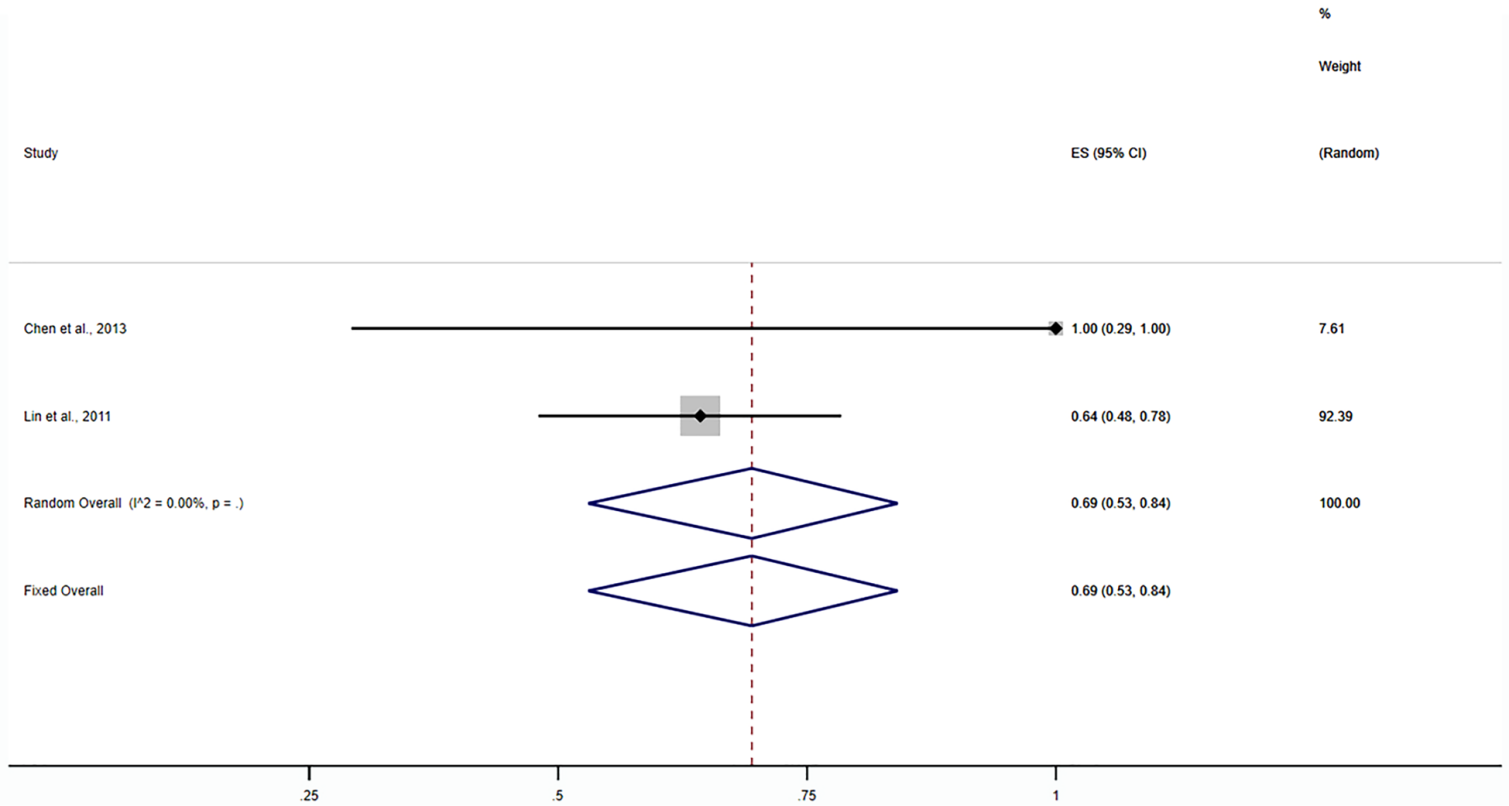
Forest plot of the ORR of the one-puncture success rate using neuronavigation

**Fig. 5:**
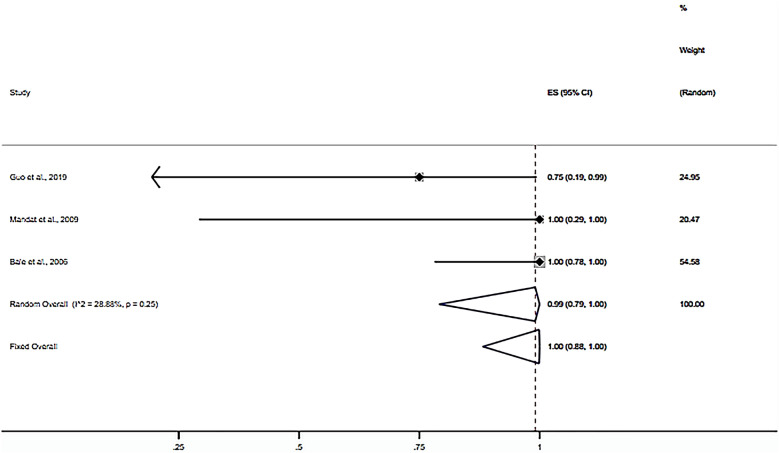
Forest plot of the ORR of the one-puncture success rate using the stereotactic technique

**Fig. 6:**
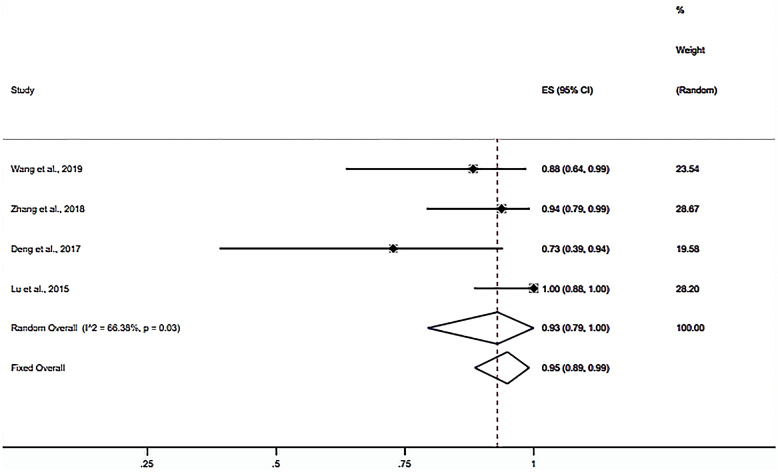
Forest plot of the ORR of the one-puncture success rate using the 3D template

Nine studies were included in calculating the one-puncture success rate (20–28). The random-effects meta-analysis results showed that the ORR of the one-puncture success rate using puncture guidance in radiofrequency thermocoagulation for TN was 92% (95% CI 0.79–1; *P* < 0.001) (Fig. 3). Subgroup analysis results showed that the ORR of neuronavigation was 69% (95% CI 0.53–0.84; *P* < 0.001) (Fig. 4). The ORR of the stereotactic technique was 99% (95% CI 0.79–1; *P* < 0.001) (Fig. 5). The ORR of the 3D template was 93% (95% CI 0.79–1; *P* < 0.001) (Fig. 6). The publication bias test results suggest no obvious publication bias (Begg’s test, *P* = 1; Egger’s test *P* = 0.77).

**Fig. 7:**
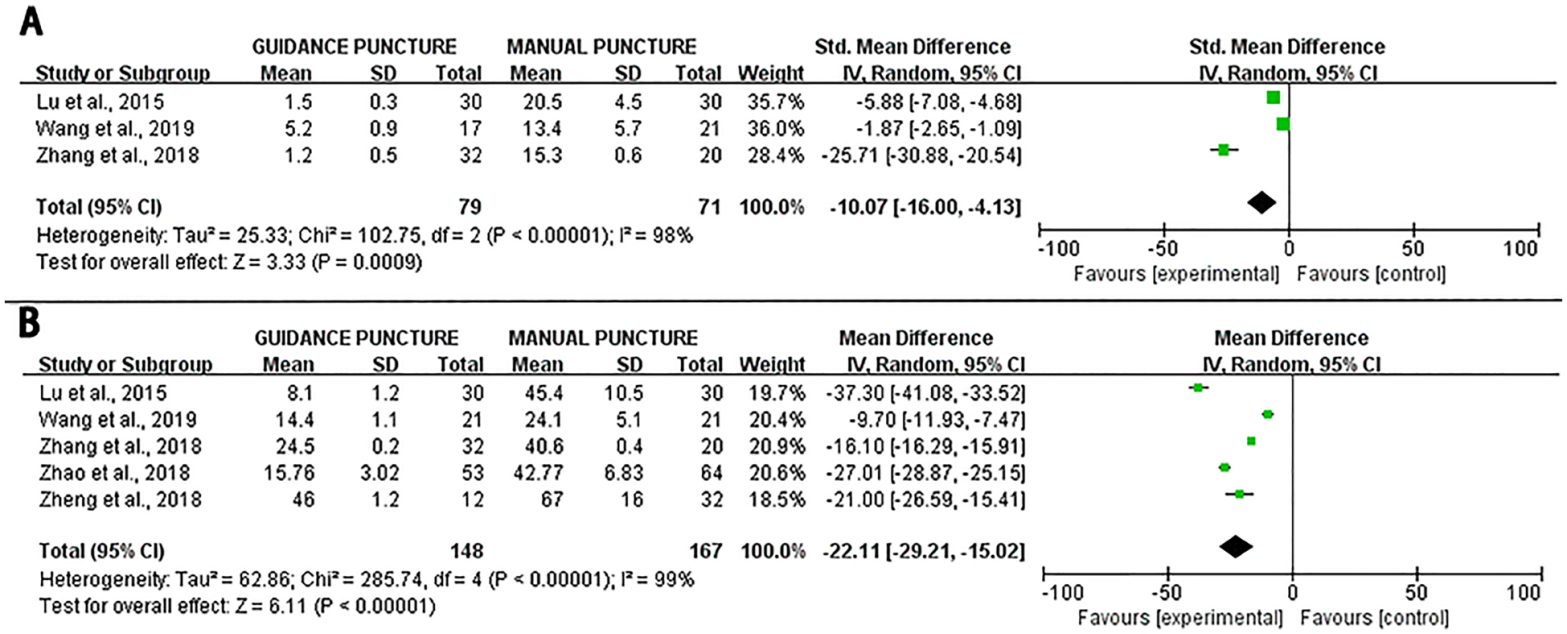
Forest plot of the cannulation time between the guided and manual puncture groups (**A**). Forest plot of the operation time between the guided and manual puncture groups (**B**)

Three studies were included in the meta-analysis comparing the cannulation time between the guided and manual puncture groups [20–22]. The results suggest a statistically significant difference in the cannulation time between the two groups (heterogeneity display, *I*^2^ = 98%; *P* < 0.001) (Fig. 7A).

Five studies were included in the meta-analysis comparing the operation time between the guided and manual puncture groups [20–24]. The results showed a statistically significant difference in the operation time between the two groups (heterogeneity display, *I*^2^ = 99%; *P* < 0.001) (Fig. 7B).

## Discussion

Neuronavigation, 3D template, and stereotactic guidance all played important roles in successfully puncturing the FO, especially in difficult-to-access FO. In three studies, patients in whom FO puncture was unsuccessful or difficult to access underwent CT navigation instead, and the one-puncture success rates were 75% [25], 75% [26], and 100% [27]. Cause the patients involved in these articles were all with difficult-to-access FO, the one-puncture success rate would be higher if consecutive patients were treated. The other studies listed in Table 1 included consecutive patients; thus, their one-puncture success rates should be higher. Few studies on manual puncture reported the one-puncture success rate and only gave the overall success rate regardless of the number of punctures. However, Zhao reported a one-puncture success rate of 25% for manual puncture [22].

The 3D template technology method achieved the shortest puncture time of approximately 1 min [20, 28], with a one-puncture success rate of 72.7–100% [20, 22, 28]. There were two studies compared 3D template with manual cannulation in one-puncture success, the results were 88.24% versus 19.05% [21] and 93.75% versus 25% [23]. Thus, this method substantially improves the puncture success rate, shortens the operation time. However, 3D templates require a long preparation time and are costly. The cannulation and operation times showed comparative significance between the groups. The results for the same guidance method varied significantly among studies due to the involvement of different surgeons with different surgical proficiencies. Therefore, we recommend using more objective indicators, such as the one-puncture success rate and puncture times, to compare puncture methods in future studies.

Stereotactic technology for puncturing the trigeminal ganglion was first reported in 1932 [15, 29]. Stereotactic techniques for TN are more commonly used in radiosurgery [30–32]. The frameless stereotactic technique demonstrated a one-puncture success rate of 100% [33, 34]. The advantage of frameless stereotactic techniques over framed stereotactic techniques is that they do not require invasive head fixation with pins, although this decreases the accuracy [35]. As the equipment has improved, the accuracy of frameless stereotactic methods has also become comparable to that of framed stereotactic methods [13, 36, 37]. A total of 18 patients were included in two frameless stereotactic technology studies [33, 34]. No studies of frameless stereotactic methods for RFT or PBC have been conducted since 2009. Whether the accuracy of frameless stereotactic methods is sufficient to puncture the FO needs further study.

All CT methods provide precise FO localization and allow simultaneous needle manipulation, although they also increase the radiation exposure to patients. CT navigation achieved one-puncture success rates of 64.3–100% [25, 27, 38–40]. The disadvantage of CT navigation is that additional time is required for registration during the operation. Compared with CT navigation, magnetic resonance navigation provides better identification of the vasculature, thereby reducing complications [41]. Chen et al. [42] introduced an electromagnetic navigation technique for guiding puncture needle placement in the FO. They did not use a control group, but they believed that electromagnetic navigation made puncture simpler, safer, faster, more accurate, and less invasive. Three studies claimed that guidance technology could reduce the cannulation time compared with manual puncture [20–22], whereas five studies claimed that guidance technology could reduce the operation time compared with manual puncture [20–24]. Regardless of which guidance technology is used, preoperative preparation does take more time. However, the intraoperative cannulation process is shorter, especially repeated cannulation and repeated verification after cannulation.

Neuronavigation technology can improve safety, reduce the incidence of complications, increase the puncture success rate, and improve the learning curve of surgeons who have only begun performing this procedure. However, muscles can bend the probe electrodes and cause major artifacts when the surgeon adjusts the direction of the needle during the puncture process. Lin et al. [38] treated 42 consecutive patients with CT navigation, and cannulation failed in 11 (26%) patients. They used CT guidance to complete the operation. In contrast to these CT techniques, 3D template, framed stereotactic, and frameless stereotactic technologies set the puncture trajectory before the operation. Neurosurgeons only need to follow the surgical plan during the operation, which can reduce reliance on the surgeon’s experience during the puncture procedure and minimize needle bending when it passes through muscles. Stereotactic technology has the highest ORR for the one-puncture success rate, followed by 3D templates and neuronavigation. However, the data are currently insufficient to confirm which guidance method has better accuracy. Neuronavigation provides superior visual–spatial information and greater operational freedom, whereas 3D template technology needs a close fit between the guide template and the face. Compared with framed stereotactic fixation, facial soft tissue fixation is far less stable than skeletal fixation. However, considering that optical systems still rely on freehand navigation, we expect that stereotactic and 3D templates will improve puncture accuracy.

In terms of the initial pain relief rate, one study achieved statistical significance [20], and one did not [23]. Although Zheng et al. [23] reported that the BNI (Barrow Neurological Institute) Pain Intensity Scale showed no difference in the initial pain relief rate, significant differences were observed in the BNI-I. In terms of the long-term pain relief rate, two studies showed statistical significance [20, 24], and one did not [23]. In terms of patient satisfaction, two studies showed statistical significance [20, 24]. In terms of postoperative complications, two studies showed statistical significance [20, 22], whereas one did not [21]. In terms of recurrence rate, one study showed statistical significance [24], and two did not [22, 23].

In the studies by Wang et al. [21] and Zhang et al. [22], 3D template technology for RFT was used for V2 TN, but the puncture target point was the foramen rotundum instead of the FO. They suggested that 3D template technology for RFT was a good choice for isolated V2 TNs via the foramen rotundum.

We found other guidance methods that are not included in Table 1. Meng et al. [43] described virtual reality-assisted RFT, although they did not record the one-puncture success rate, puncture time, or operation time. Tsai et al. [44] used intraoperative CT with magnetic resonance image fusion to guide RFT. This method improved the 2 year pain relief and avoided puncture-related complications. Brandmeir et al. [45] reported a case of robot-assisted stereotactic PBC. However, they did not record the one-puncture success rate, puncture time, or operation time.

Accurate puncture positioning improves patient satisfaction. However, no consensus has been reached with regard to postoperative complications, recurrence rate, initial pain relief rate, or long-term efficacy. With the help of various auxiliary puncture systems, puncture approaches at different angles are no longer dependent on surgeons’ skill and experience. Individualized and customized surgical plans can greatly reduce the difficulty of surgery. Accurate cannulation can reduce the temperature and duration of radiofrequency thermocoagulation and achieve better treatment results. Considering that optical systems still rely on freehand navigation, we believe these results are reasonable. Due to the specificity of foramen ovale puncture and the extremely low tolerance for target site selection, we expect that stereotactic and 3D templates will improve puncture accuracy.

## Limitation

Main weakness of these results is that none of the studies are comparing any two methods. This means that all the surgeons are different from each other and surgeon experience is one of the key determinants of the one-puncture success rate, operation and cannulation time. In this systematic review, 11 studies were analyzed; however, for subgroup analysis, the results seems weak. There may have been reporting bias or incomplete retrieval or inadvertent exclusion of relevant studies.

## Conclusions

Neuronavigation, 3D template, and stereotactic guidance all have an absolute advantage in assisting in puncturing the FO, can improve the one-puncture success rate, the learning curve, and safety and reduce the incidence of complications and the puncture, cannulation, and operation times. The accuracies of the one-puncture success rate (ORR) of the stereotactic technique, 3D template and neuronavigation were 99%, 93% and 69%, respectively. In any case, RFT with puncture guidance is a good currently available treatment option, especially for difficult-to-access FO.

## Data Availability

Not applicable.

## Abbreviations

TN: Trigeminal neuralgia
RFT: Radiofrequency thermocoagulation
GR: Glycerol rhizolysis
MVD: Microvascular decompression
PBC: Percutaneous balloon compression
ORR: Objective response rate
FO: Foramen ovale
RCTs: Randomized controlled trials
BNI: Barrow Neurological Institute

## References

[1] Zakrzewska JM, Linskey ME (2014). Trigeminal neuralgia. BMJ.

[2] Ran B, Wei J, Zhong Q, Fu M, Yang J, Chen X (2019). Long-term follow-up of patients treated with percutaneous radiofrequency thermocoagulation via the foramen rotundum for isolated maxillary nerve idiopathic trigeminal neuralgia. Pain Med.

[3] Scranton RA, Shah K, Cohen-Gadol AA (2019). Alternative customized instrumentation and technique for percutaneous balloon compression rhizotomy for trigeminal neuralgia. J Neurosurg.

[4] Noorani I, Lodge A, Vajramani G, Sparrow O (2019). The effectiveness of percutaneous balloon compression, thermocoagulation, and glycerol rhizolysis for trigeminal neuralgia in multiple sclerosis. Neurosurgery.

[5] Bendtsen L, Zakrzewska JM, Heinskou TB, Hodaie M, Leal PRL, Nurmikko T (2020). Advances in diagnosis, classification, pathophysiology, and management of trigeminal neuralgia. Lancet Neurol.

[6] Wang JY, Bender MT, Bettegowda C (2016). Percutaneous procedures for the treatment of trigeminal neuralgia. Neurosurg Clin N Am.

[7] Réthi A (1913). Die elektrolytische Behandlung der Trigeminusneuralgien. Munch Med Wochenschr.

[8] Kanpolat Y, Savas A, Bekar A, Berk C (2001). Percutaneous controlled radiofrequency trigeminal rhizotomy for the treatment of idiopathic trigeminal neuralgia: 25-year experience with 1,600 patients. Neurosurgery.

[9] Xue TQ, Zhang QX, Bian H, Zhou PC, Liu C, Niu SF (2019). Radiofrequency thermocoagulation through foramen rotundum versus foramen ovale for the treatment of V2 trigeminal neuralgia. Pain Physician.

[10] Koning MV, Koning NJ, Koning HM, van Kleef M (2014). Relationship between sensory stimulation and side effects in percutaneous radiofrequency treatment of the trigeminal ganglion. Pain Pract.

[11] Erdine S, Ozyalcin NS, Cimen A, Celik M, Talu GK, Disci R (2007). Comparison of pulsed radiofrequency with conventional radiofrequency in the treatment of idiopathic trigeminal neuralgia. Eur J Pain.

[12] Hamid AI, Qureshi AA, Bhatti IH (1993). Percutaneous radiofrequency retrogasserian rhizotomy for trigeminal neuralgia. J Pak Med Assoc.

[13] Steinmeier R, Rachinger J, Kaus M, Ganslandt O, Huk W, Fahlbusch R (2000). Factors influencing the application accuracy of neuronavigation systems. Stereotact Funct Neurosurg.

[14] Xue T, Yang W, Guo Y, Yuan W, Dai J, Zhao Z (2015). 3D image-guided percutaneous radiofrequency thermocoagulation of the maxillary branch of the trigeminal nerve through foramen rotundum for the treatment of trigeminal neuralgia. Medicine (Baltimore).

[15] Kirschner M (1932). Electrocoagulation des ganglion gasseri. Zentralbl Chir.

[16] Waltregny AJ (1982). A stereotactic frame for trigeminal ganglionectomy. Appl Neurophysiol.

[17] Liberati A, Altman DG, Tetzlaff J, Mulrow C, Gøtzsche PC, Ioannidis JPA (2009). The PRISMA statement for reporting systematic reviews and meta-analyses of studies that evaluate health care interventions: explanation and elaboration. PLoS Med.

[18] Higgins JPT, Altman DG, Gøtzsche PC, Jüni P, Moher D, Oxman AD (2011). The Cochrane Collaboration’s tool for assessing risk of bias in randomised trials. BMJ.

[19] Sterne JA, Hernán MA, Reeves BC, Savović J, Berkman ND, Viswanathan M (2016). ROBINS-I: a tool for assessing risk of bias in non-randomised studies of interventions. BMJ.

[20] Li-Juan LU, Han Y, Hong-Bo H, Xie H (2015). 3D printing puncture navigation module-guided percutaneous radiofrequency thermocoagulation for treatment of trigeminal neuralgia. Chin J Pain Med.

[21] Wang R, Han Y, Lu L (2019). Computer-assisted design template guided percutaneous radiofrequency thermocoagulation through foramen rotundum for treatment of isolated V2 trigeminal neuralgia: a retrospective case-control study. Pain Res Manag.

[22] Zhang LG, Deng MH, Long X, Wang ZZ (2018). 3D printing navigation template-guided percutaneous radiofrequency thermocoagulation for V2 trigeminal neuralgia treatment. Hua Xi Kou Qiang Yi Xue Za Zhi.

[23] Zheng XB, Gao ZW, Mo HB, Lin Q, Wang HQ, Yu LH (2018). Neuronavigation-assisted percutaneous radiofrequency thermocoagulation of trigeminal gasserian ganglion for refractory craniofacial pain. Zhonghua Yi Xue Za Zhi.

[24] Zhao S, Deng M, Cai H, Meng Q, Fang W, Ke J (2018). Clinical efficacy evaluation for treating trigeminal neuralgia using a personalized digital guide plate-assisted temperature-controlled radiofrequency. J Craniofac Surg.

[25] Bohnstedt BN, Tubbs RS, Cohen-Gadol AA (2012). The use of intraoperative navigation for percutaneous procedures at the skull base including a difficult-to-access foramen ovale. Neurosurgery.

[26] Guo Z, Wang Z, Li K, Du C, Zhao X, Cheng M (2019). Unconventional facial entry points confirmed using a 3D CT reconstruction-guided stereotactic approach to radiofrequency thermocoagulation for the treatment of trigeminal neuralgia: a series of case reports. Pain Med.

[27] Georgiopoulos M, Ellul J, Chroni E, Constantoyannis C (2014). Minimizing technical failure of percutaneous balloon compression for trigeminal neuralgia using neuronavigation. ISRN Neurol.

[28] Deng M, Cai H, Fang W, Long X (2018). Three-dimensionally printed personalized guide plate for percutaneous radiofrequency thermal coagulation in idiopathic trigeminal neuralgia. Int J Oral Maxillofac Surg.

[29] Kirschner M (1933). Die punktionstechnik und die elektrokoagulation des ganglion gasseri. Arch Klin Chir.

[30] Wang AP, Suryavanshi T, Marcucci M, Fong C, Whitton AC, Reddy KKV (2020). Radiation necrosis following stereotactic radiosurgery for trigeminal neuralgia. Can J Neurol Sci.

[31] Helis CA, Hughes RT, Munley MT, Bourland JD, Jacobson T, Lucas JT (2020). Results of a third Gamma Knife radiosurgery for trigeminal neuralgia. J Neurosurg.

[32] Mohammed N, Hung YC, Muttikkal TJE, Bliley RC, Xu Z, Sheehan JP (2019). Changes in the muscles of mastication before and after primary stereotactic radiosurgery in patients with idiopathic trigeminal neuralgia. J Neurosurg.

[33] Bale RJ, Laimer I, Martin A, Schlager A, Mayr C, Rieger M (2006). Frameless stereotactic cannulation of the foramen ovale for ablative treatment of trigeminal neuralgia. Neurosurgery.

[34] Mandat T, Brozyna B, Krzymanski G, Podgorski JK (2009). An image-guided, noninvasive method of cannulation of the foramen ovale for awake, percutaneous radiofrequency rhizotomy. J Neurosurg.

[35] Grimm F, Naros G, Gutenberg A, Keric N, Giese A, Gharabaghi A (2015). Blurring the boundaries between frame-based and frameless stereotaxy: feasibility study for brain biopsies performed with the use of a head-mounted robot. J Neurosurg.

[36] Holloway KL, Gaede SE, Starr PA, Rosenow JM, Ramakrishnan V, Henderson JM (2005). Frameless stereotaxy using bone fiducial markers for deep brain stimulation. J Neurosurg.

[37] Dorward NL, Alberti O, Palmer JD, Kitchen ND, Thomas DG (1999). Accuracy of true frameless stereotaxy: in vivo measurement and laboratory phantom studies technical note. J Neurosurg.

[38] Lin MH-C, Lee M-H, Wang T-C, Cheng Y-K, Su C-H, Chang C-M (2011). Foramen ovale cannulation guided by intra-operative computed tomography with integrated neuronavigation for the treatment of trigeminal neuralgia. Acta Neurochir (Wien).

[39] Ding W, Chen S, Wang R, Cai J, Cheng Y, Yu L (2016). Percutaneous radiofrequency thermocoagulation for trigeminal neuralgia using neuronavigation-guided puncture from a mandibular angle. Medicine (Baltimore).

[40] Aydoseli A, Akcakaya MO, Aras Y, Sabanci PA, Unal TC, Sencer A (2015). Neuronavigation-assisted percutaneous balloon compression for the treatment of trigeminal neuralgia: the technique and short-term clinical results. Br J Neurosurg.

[41] Lepski G, Mesquita Filho PM, Ramina K, Bisdas S, Ernemann U, Tatagiba M (2015). MRI-based radiation-free method for navigated percutaneous radiofrequency trigeminal rhizotomy. J Neurol Surg A Cent Eur Neurosurg.

[42] Chen M-J, Gu L-X, Zhang W-J, Yang C, Dong M-J (2013). Electromagnetic navigation-guided radiofrequency thermocoagulation in trigeminal neuralgia: technical note with three case reports. J Neurol Surg A Cent Eur Neurosurg.

[43] Meng F-G, Wu C-Y, Liu Y-G, Liu L (2009). Virtual reality imaging technique in percutaneous radiofrequency rhizotomy for intractable trigeminal neuralgia. J Clin Neurosci.

[44] Tsai P-J, Lee M-H, Chen K-T, Huang W-C, Yang J-T, Lin MH-C (2019). Foramen ovale cannulation guided by intraoperative computed tomography with magnetic resonance image fusion plays a role in improving the long-term outcome of percutaneous radiofrequency trigeminal rhizotomy. Acta Neurochir (Wien).

[45] Brandmeir NJ, Sather MD (2017). A technical report of robot-assisted stereotactic percutaneous rhizotomy. Pain Med.

